# Intravascular Ultrasound Guidance for Transcatheter Pulmonary Valve Replacement: A Feasibility Study

**DOI:** 10.1016/j.jacadv.2026.103309

**Published:** 2026-10-06

**Authors:** Karol A. Sadowski, Michał Kania, Wiktor Stark, Olgierd Woźniak, Yusuke Kobari, Jacek Kądziela, Gary S. Mintz, Ole De Backer, Marcin Demkow, Łukasz Kalińczuk

**Affiliations:** a National Institute of Cardiology, Warsaw, Poland; b Københavns Universitet, The Heart Center–Rigshospitalet, Copenhagen, Denmark; c Cardiovascular Research Foundation, New York, New York, USA

**Keywords:** geometric orifice area, procedural imaging, right ventricular outflow tract, stent-frame expansion

**Figure:**
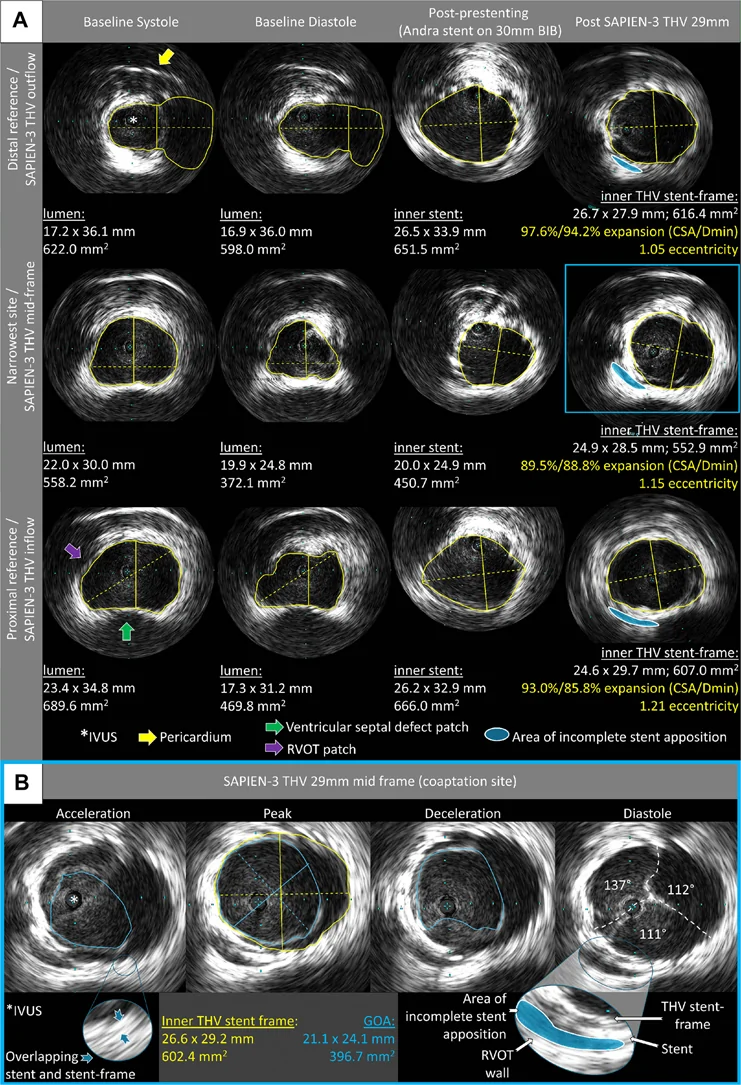

**What is the clinical question being addressed?**

Can intraprocedural large-field IVUS provide tomographic assessment of RVOT and valve geometry during TPVR?

**What is the main finding?**

IVUS revealed focal circularization, subnominal valve expansion, and a leaflet-defined GOA associated with follow-up gradients.

Transcatheter pulmonary valve replacement (TPVR) is used in the treatment of right ventricular outflow tract (RVOT) or conduit dysfunction and failed surgical bioprostheses. Computed tomography guides sizing but cannot provide intraprocedural feedback. Projection angiography cannot provide a true short-axis assessment of the RVOT geometry, achieved transcatheter heart valve (THV) expansion, or leaflet-defined geometric orifice area (GOA). Large-field 0.035-inch intravascular ultrasound (IVUS) provides an intraluminal tomographic perspective during structural valve interventions.^1^ We assessed IVUS-derived RVOT geometry, THV expansion, and GOA vs angiography and echocardiographic follow-up data.

This retrospective single-center analysis used prospectively collected records. The study protocol complied with the Declaration of Helsinki and was approved by the Institutional Review Board of the National Institute of Cardiology, Warsaw, Poland. Written informed consent was obtained from all patients. Landing-zone prestenting was followed by the implantation of a 22-mm Melody THV (Medtronic) or 29-mm SAPIEN 3 THV (Edwards Lifesciences), deployed nominally or with 4-mL overfilling. IVUS (10-MHz, 8.5-F Visions PV 0.035-inch catheter; Philips Volcano) was performed at baseline, after prestenting, and after THV implantation over a 0.035-inch stiff procedural guidewire. A single reader blinded to angiographic and echocardiographic outcomes performed an offline analysis. Cross-sectional area (CSA), minimal diameter (D_min_), and maximal diameter (D_max_) were measured at proximal, narrowest, and distal levels; post-THV implantation levels were inflow, midframe (coaptation), and outflow. Peak-systolic GOA, commissural angles, and pinwheeling were evaluated. Serial angiographic diameters (D_angio_) were measured in the best projection. Expansion used device- and deployment-specific reference dimensions. Follow-up gradients and pulmonary insufficiency data were obtained from clinically indicated echocardiography; median follow-up duration was 271 (95-721) days, and follow-up mean gradient was the per-patient mean of available postdischarge measurements. Data are provided as mean ± SD or median [Q1-Q3]. Paired comparisons used paired *t* or Wilcoxon tests according to Shapiro-Wilk; independent skewed groups used Mann-Whitney *U*. Pearson/Spearman correlations included Fisher-z 95% CIs. Tests were 2-sided; *P* < 0.05 was considered significant (PASW Statistics 18, IBM).

Among 35 TPVR procedures performed between February 2018 and December 2021, IVUS was used in 16 (45.7%), based on catheter availability and the operator’s willingness to modestly prolong the procedure. Patients were 30.4 ± 6.9 years old; 6 of 16 (37.5%) were women; 12 of 16 (75.0%) received SAPIEN 3 valves; and 4 of 16 (25.0%) received Melody valves. Most had surgically modified RVOTs; tetralogy of Fallot was present in 14 of the 16 (87.5%). All 16 (100%) had significant pulmonary regurgitation, and 7 of 16 (43.8%) also had significant pulmonary stenosis. Balloon interrogation was performed in all 16 (100%); therapeutic predilatation was performed in 3 of 16 (18.8%).

Compared with D_angio_, baseline IVUS D_min_ was 0.6 mm (2.6%) smaller proximally, 2.7 mm (13.2%) smaller at the narrowest site, and 0.4 mm (1.8%) smaller distally, whereas D_max_ was 7.8 mm (33.5%), 1.9 mm (9.3%), and 4.9 mm (21.5%) larger, respectively. Differences were significant for D_min_ at the narrowest site and D_max_ at both reference levels.

Paired baseline-to-post-THV comparisons showed angiographic enlargement proximally (23.3 ± 4.2 to 27.1 ± 5.7 mm; *P* = 0.030), at the narrowest site (20.5 ± 4.5 to 26.1 ± 6.4 mm; *P* = 0.001), and distally (22.8 ± 5.0 to 27.7 ± 5.8 mm; *P* = 0.002); post-prestenting values were 23.8 ± 5.0, 22.6 ± 5.5, and 24.6 ± 5.0 mm, respectively. IVUS localized remodeling to the narrowest site: D_min_ increased from 17.8 ± 4.0 to 23.9 ± 4.5 mm, D_max_ from 22.4 ± 4.5 to 26.8 ± 5.6 mm, and CSA from 320.5 ± 124.3 to 517.5 ± 167.6 mm^2^, while D_max_/D_min_ decreased from 1.3 ± 0.1 to 1.1 ± 0.1. Proximal and distal reference CSAs did not increase significantly, and the average of the 3 level-specific mean D_max_ values was unchanged (27.1 vs 27.0 mm).

Post THV implantation, D_angio_ correlated more strongly with D_min_ than with D_max_ at inflow (*r* = 0.861 [95% CI: 0.637-0.951] vs 0.551 [0.076-0.822]), midframe (0.862 [0.640-0.951] vs 0.626 [0.189-0.856]), and outflow (0.850 [0.612-0.947] vs 0.804 [0.513-0.929]). Among 12 SAPIEN 3 recipients, midframe CSA expansion was 88.5% ± 12.1% after nominal deployment (4/12) and 84.8% ± 10.8% with 4-mL overfilling (8/12); Melody midframe CSA expansion was 82.4% ± 8.6% (4/16). CSA-derived expansion remained <90% at inflow, midframe, and outflow (84.9% ± 12.7%, 85.9% ± 16.5%, and 84.8% ± 14.4%, respectively). D_min_ was 10.1% to 16.1% smaller than nominal (all *P* < 0.001), whereas D_max_ was similar to nominal at inflow and midframe and 1.2 mm smaller at outflow (*P* = 0.068).

SAPIEN 3 and Melody GOA were 3.89 ± 0.55 and 1.82 ± 0.46 cm^2^, respectively, representing 65.1% ± 9.3% vs 77.6% ± 9.4% of measured coaptation-site frame CSA and 58.0% ± 6.7% vs 50.6% ± 12.9% of deployment-specific reference CSA (Figure 1). Maximum and minimum commissural angles were 136° ± 10° and 106° ± 9° (range: 30° ± 18°); pinwheeling occurred in 1 of 16 (6.2%). Follow-up mean gradient averaged 12.0 ± 5.7 mm Hg. GOA area, D_max_, and D_min_ correlated inversely with this gradient (Spearman ρ = −0.700 [95% CI: −0.888 to −0.313], −0.688 [−0.883 to −0.292], and −0.665 [−0.873 to −0.253], respectively; all *P* ≤ 0.005); these associations were exploratory. Trace/mild pulmonary insufficiency occurred in 8 of 16 (50.0%) and was unrelated to THV expansion. Among 10 patients with paired baseline/post-prestenting IVUS, proximal D_max_ change was −3.93 [−6.94 to −2.90] mm in 4 of 10 with trace/mild paravalvular leak (PVL) vs +2.07 [1.34-2.79] mm in 6 of 10 without leak (*P* = 0.067).

**Figure 1:**
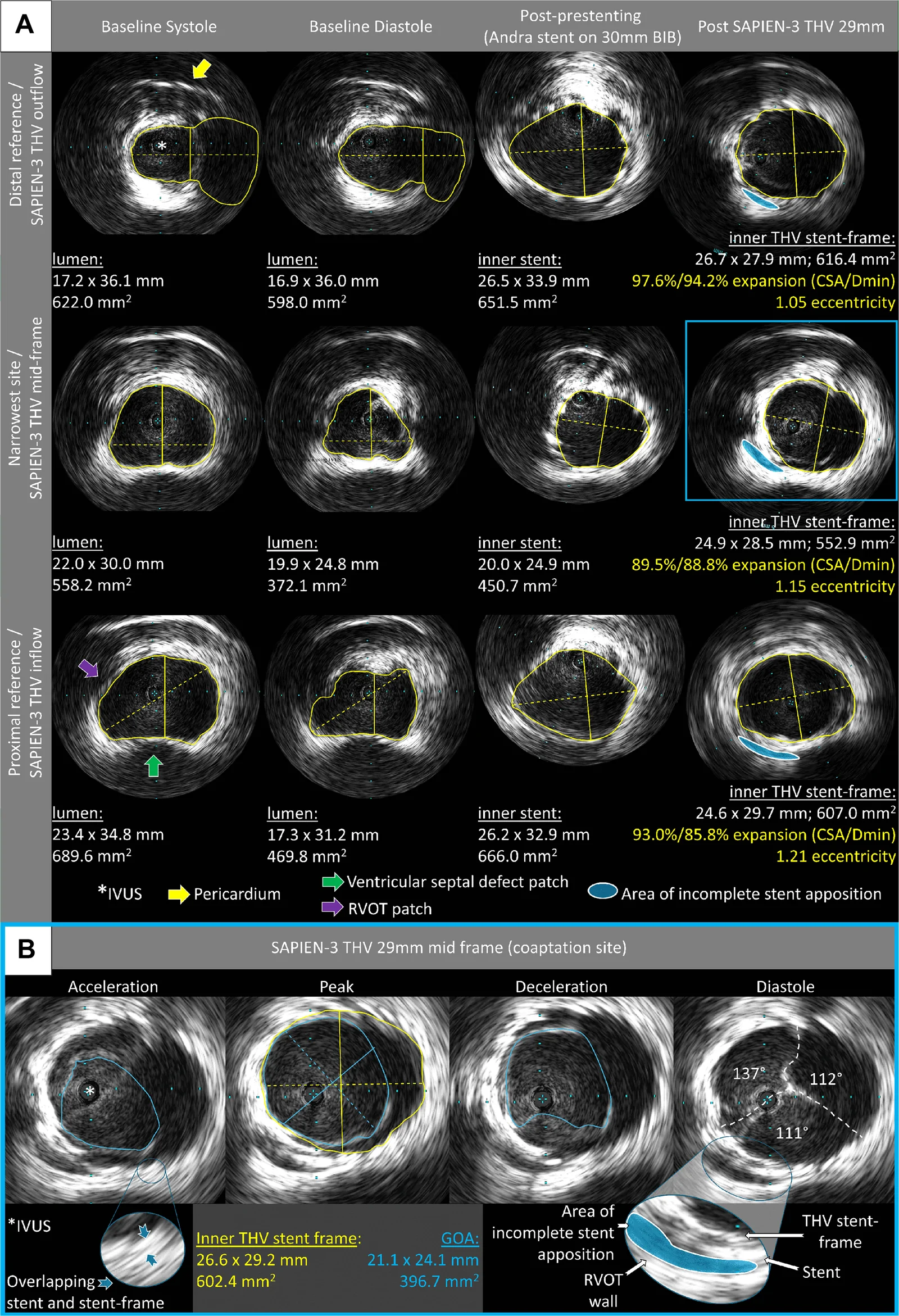
Serial IVUS Assessment During Transcatheter Pulmonary Valve Replacement (A) Representative matched short-axis IVUS sections at the distal reference/outflow, narrowest site/midframe, and proximal reference/inflow before intervention, after prestenting, and after SAPIEN 3 implantation, demonstrating focal remodeling and incomplete apposition. The illustrated 29-mm SAPIEN 3 valve was deployed with 1-mL underfilling; panel measurements are patient-specific, whereas cohort expansion metrics are reported in the text. (B) Midframe IVUS across the leaflet cycle with systolic GOA planimetry. Blue outlines indicate incomplete apposition. BIB = balloon-in-balloon catheter; CSA = cross-sectional area; GOA = geometric orifice area; IVUS = intravascular ultrasound; RVOT = right ventricular outflow tract; THV = transcatheter heart valve.

IVUS provided segment-specific information not apparent on angiography. Although angiography suggested generalized landing-zone enlargement, IVUS showed that TPVR principally relieved a focal narrowest-site constraint through increased D_min_ and reduced eccentricity rather than uniformly enlarging the RVOT; a satisfactory projected diameter may therefore coexist with a constrained smallest dimension.

Achieved THV expansion was consistently subnominal. Underexpansion and deformation have been associated with adverse hemodynamics, altered leaflet mechanics, hypoattenuated leaflet thickening, and later valve deterioration. D_max_ approached nominal while D_min_ remained 10% to 16% smaller, and CSA expansion was approximately 85%; D_max_ alone could conceal eccentric area underexpansion. Leaflet-defined GOA was substantially smaller than frame area and related to follow-up gradient.

Global underexpansion, focal residual narrowing, and incomplete apposition may require different responses. Post-THV IVUS may help identify a constrained frame requiring further optimization; the PVL finding remains exploratory.

In an unpublished companion bench analysis, marked noncoaxiality—mean angulation 24.6° ± 10.0°—inflated D_max_ and CSA by approximately 10% to 12%, whereas D_min_ remained robust.^2^ The wire-tracking catheter is not self-centering, and oblique acquisition may inflate D_max_ and CSA or reduce contour completeness; D_min_ is therefore the most defensible geometric anchor when the catheter cannot remain centered or coaxial. IVUS and intracardiac echocardiography are complementary: Intracardiac echocardiography provides global visualization, Doppler, and regurgitation localization, whereas IVUS can traverse the landing zone and deployed THV to obtain anatomically matched short-axis sections. The 10-MHz platform has limited near-field definition for subtle leaflet edges and small apposition gaps; future devices should combine improved centering, higher-frequency imaging, and intraluminal Doppler.

Our findings support geometry-guided TPVR using D_min_, level-specific CSA expansion, apposition, and GOA, but intervention thresholds cannot be derived from this small series. Limitations include a retrospective single-center design, small sample, selective IVUS use, heterogeneous devices, single-reader analysis without reproducibility testing or a core laboratory, catheter noncentering, qualitative pinwheeling assessment, and limited near-field resolution. The PVL analysis was underpowered.

## Funding support and author disclosures

This work was supported by the statutory grant of the National Institute of Cardiology, Warsaw, Poland (no. 2.15/VI/26). The authors have reported that they have no relationships relevant to the contents of this paper to disclose.
